# Influence of Klein edges on Phononic and electronic transport in circular graphene devices

**DOI:** 10.1038/s41598-024-80746-y

**Published:** 2024-11-25

**Authors:** M. Amir Bazrafshan, Farhad Khoeini, Bartłomiej Szafran

**Affiliations:** 1 Department of Physics, University of Zanjan, P.O. Box 45195-313, Zanjan, Iran; 2 Faculty of Physics and Applied Computer Science, AGH University of Krakow, al. Mickiewicza 30, Krakow, 30-059 Poland

**Keywords:** Klein edge, Electronic transport, Phononic transport, Graphene, Nanoring, Nanodisk, Materials science, Nanoscience and technology, Physics

## Abstract

We study the electron and phonon transport coefficients of graphene disks and rings in the presence of Klein edges. We examine the transport characteristics by changing of the outer and inner radius using the non-equilibrium Green’s function approach. We find that the effect of the nanodisk radius is highly influenced by the Klein edges, such that at small radii, armchair Klein edges can help preserve the electronic transport coefficient from suppression, while zigzag Klein edges significantly suppress the transmission spectrum, highlighting the importance of the edge atom sublattice. The behavior is also observed in cases where only one side of the circular disk is preserved, showing that it is not rooted in the symmetric geometry of the circle. The value of the outer radius has a more regular influence on the electronic conductance than the value of the inner one. However, in the examined sizes, the phononic spectrum does not exhibit a clear dependence on the edges. Our results contribute to the understanding of the behavior of Klein edges, which is crucial for the design of high-performance nanoscale electronic devices, the creation of stable qubits for advances in quantum computing.

**Supplementary Information:**

The online version contains supplementary material available at 10.1038/s41598-024-80746-y.

## Introduction

Electronic elements, including transistors in integrated circuits, are now nanoscale. Conducting elements based on carbon nanotubes^1^and graphene^2^have been extensively studied in last decades. Besides, graphene’s outstanding properties^3^, its stability in the conventional environment makes it an excellent candidate to explore the nanoscale world^4^. Recently, carbon based semiconducting structures such as C_2_N^5,6^, and C_3_N^7^ are realized experimentally.

It is possible to fabricate graphene nanoribbons with atomic precision^4,8^. Due to the quantization of electron’s momentum in quasi-one-dimensional materials, graphene nanoribbons can possess versatile electronic gap sizes^9–11^, depending on the ribbon edge and the number of atoms in an elementary cell. In the simple tight-binding (TB) approximation, the zigzag edge states are responsible for the metallicity of zigzag graphene nanoribbons (ZGNRs)^9–11^. In these ribbons and close to the Fermi energy, the electronic states are highly localized at the edges and backscattering is inhibited by the valley conservation mechanism^9,11^. The electronic properties of graphene near the Fermi energy are dominated by the $\:{p}_{z}$orbital^9,12,13^, making it convenient for theoretical study using approximations such as the tight-binding method. Since geometry plays a crucial role at the nanoscale, the study of circular geometries, which contains different edge chiralities, is an interesting venue to explore. Klein edges, which are single bonded atoms, like other defects, can reduce the mean free path and cause the dominance of the Anderson localization regime^14^. However, the backscattering efficiency of a transport system is related to how close the electronic configurations of the device and the leads are. The behavior of the electronic transport properties is particularly important in the field of thermoelectrics, where keeping the electronic transport coefficient untouched and degrading the phononic transport coefficient is considered a promising way to improve the thermoelectric efficiency.

Edge imperfections in graphene are experimentally observed with an atomic precision, specifically the presence of unsaturated single bonded carbon atoms is confirmed^15–17^. In addition, the hydrogenation of the armchair graphene nanoribbons (AGNRs) can be experimentally controlled, as reported in^18^. Furthermore, the fabrication of graphene nanorings and nanodisks with a minimum radius of 50 nm is reported in^19^. Another work, Ref^20^, reports the synthesis of graphene nanodisks with a well-defined circular geometry and a diameter of ~ 40 nm, exhibiting an energy gap of 0.73 eV. However, ring structures are mainly chosen to study the Aharonov-Bohm effect^21–24^.

Klein edges along both sides of a ZGNR are predicted to be magnetic, regardless of hydrogen passivation^25,26^. These single carbon bonds can last ~ 2 s before going under edge reconstruction^16^. In addition, there are experimental reports showing that Klein edges along the armchair direction are more stable under electron irradiation than those along the zigzag direction^27^. Theoretical analysis suggests that modifying the edges of a nanoribbon with hydrogen can result in the appearance of single-bonded atom features^18^. This shows that it is possible to overcome the edge instability and utilize their unique characteristics. Single bonded carbon atoms in ZGNRs are located on different sublattices, while in AGNRs they can be located on either the same or different sublattices^18,28^. ZGNRs with Klein edges also possess dispersive bands in the electronic structure due to the different occurrence of Klein edge atom sublattices, but AGNRs with Klein edge atoms on the same sublattice produce flat bands, showing that unpaired electrons are highly localized regardless of the AGNR width^18^. Ref^29^ reports that nanocrystalline graphene spirals (nc-G) with enhanced electrical conductivity are experimentally fabricated with Klein edges, showing fast progress in the fabrication of circular shape geometries with high precision.

Using the tight-binding and four nearest neighbor (4NN) force-constant (FC) approaches implemented in the non-equilibrium Green’s function (NEGF) method, we study the electronic and phononic transport properties of a circular graphene ring with varying inner and outer radius connected to ZGNR electrodes. However, the tight-binding and FC approximations for studying defected graphene is appropriate to gain insight into defected graphene nanostructures^30–32^. The numerical results indicate that the nature of the transport system is mainly determined by the edge geometry of a nanodisk (or a ring with an inner radius of zero). The sublattices involved in the device play a crucial role in determining the behavior of electrons. We have not observed a meaningful change in the phonon-related properties of the systems due to the long-range interaction of phonons. However, controlling the disk size can change the sensitivity of the disk surface to phononic properties. When there are no Klein edges present along the zigzag direction (zKLs), the Klein edges located along the armchair direction (aKLs) of the system can help recover the transmission spectrum of the system to that of the electrodes. However, phonons do not exhibit a similar behavior, demonstrating the exceptional capability of these edge geometries for engineering the transport coefficient of both electrons and phonons.

While the precise geometries explored in this work have not yet been realized experimentally, rapid advances in nanofabrication techniques and atomic-scale manipulation make such structures increasingly feasible. The structures are simulated in their free-standing form. This implies that the verification of the predicted physical behavior reported here must be done in a way that preserves the integrity of the fabricated structure close to the free-standing form, which is an important challenge. However, as reported in^18^, edge modification of graphene nanoribbons with additional hydrogen atoms can change the electronic configuration of the system to resemble the presence of Klein edges.

In the following, we describe the model and the method in the next section, then discuss the effect of the outer radius and the inner radius in two consecutive subsections of section three. Some test models are then examined in the last subsection of section three. Finally, we conclude our study in the last section.

## Model and method

**Fig. 1:**
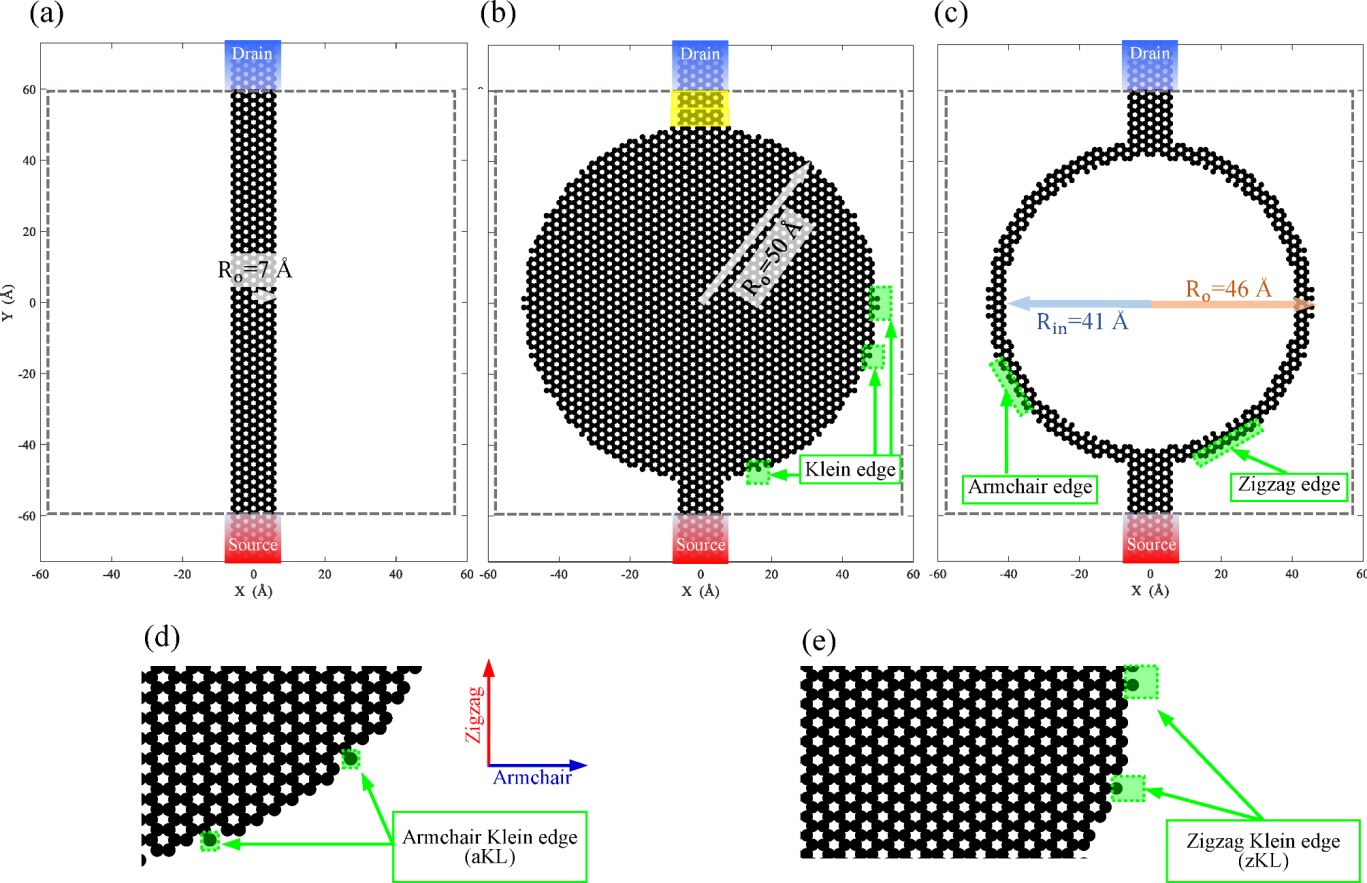
(**a**) Starting point structure ($\:{\text{R}}_{\text{o}}$=7 Å) corresponding to the width of electrodes. (**b**) A nanodisk with an outer radius of 50 Å. (**c**) One of the selected structures to study with $\:{\text{R}}_{\text{o}}$=46 Å and $\:{\text{R}}_{\text{i}\text{n}}$=41 Å. Examples of Klein, armchair and zigzag edge geometries are shown by green boxes. (d) and (e), zoom-in pieces of the outer edge of the device, including the armchair and zigzag Klein edges, respectively. Drain and source electrodes are shown by blue and red gradient boxes. The device part of the transport system is indicated by dashed rectangle. Note that the size of the scattering region is 120 Å for all studied structures. The transport direction is considered along Y direction.

We consider a nanodisk or a nanoring defined within a metallic zigzag nanoribbon that serves as source and drain electrodes connected to the scattering region. The starting point is a zigzag nanoribbon with a width of 12 atoms and a length of 120 Å (Fig. 1 (a)), which is slightly larger than the largest outer radius studied in this work. We study systems with the radius increased up to 50 Å in steps of 1 Å (Fig. 1 (b)). See Supplementary Material (SM) for details on edge geometry (Figure S.1). The device region lies within the dashed rectangle shown in Fig. 1. According to the figure, the device (scattering region) includes at least two unit cells of each electrode, represented by yellow transparent rectangles in Fig. 1(b).

Two source/drain electrodes are connected to provide and gather electrons to/from the device. The outer radius is represented by $\:{\text{R}}_{\text{o}}$ and the inner radius by $\:{\text{R}}_{\text{i}\text{n}}$. We set the one-dimensional width of the rings to $\:{\text{R}}_{\text{o}}-{\text{R}}_{\text{i}\text{n}}\ge\:\:5\:\text{Angstrom:}$, e.g. for $\:{\text{R}}_{\text{o}}$=46 Å the inner radius is varied up to $\:{\text{R}}_{\text{i}\text{n}}$=41 Å (Fig. 1(c)).

For convenience, we refer to all types of dangling bonds as Klein edges. Taking the transport direction of the system as a reference, if a Klein edge is located along the armchair direction, we call it an aKL (Fig. 1(d)), likewise, a Klein edge located along the zigzag direction is called a zKL (Fig. 1(e)).

The electronic properties are studied using the tight-binding Hamiltonians implemented in the NEGF formalism. The Hamiltonians in the TB approximation are given by^33^

1 $$H={\sum\:}_{i}{\epsilon\:}_{i}{c}_{i}^{\dag }{c}_{i}{\sum\:}_{lt;i,jgt;}{t}_{i,j}{c}_{i}^{\dag }{c}_{i}+\text{H}.\text{c}.,$$

where $\:{\epsilon\:}_{i}$ is the on-site energy, and $\:{t}_{i,j}$ is the hopping parameter between atoms *i* and *j*. Also, $c_{i}^{\dag }$ $\:{c}_{i}$are the creation and annihilation operators of electrons. The TB parameters for graphene are taken from^34^, and are also listed in Table 1. Details of the calculation method are given in^35^.

We would like to emphasize that the first-order tight-binding approximation refers to a simplified model to describe the behavior of electrons in a material. This approximation focuses on the most basic interactions, ignoring more complex phenomena such as electron-electron interactions and thermal effects. Essentially, it’s a useful tool for gaining insights into the electronic properties of materials, serving as a fundamental step before incorporating more detailed factors.

The inclusion of the spin-orbit interaction, the substrate effect, the electron-electron interaction, and the thermal effect can be key factors that could improve the accuracy of the approximations. However, the spin-orbit coupling is negligible in graphene^36^. Since our focus is on the physics of the Klein edges, and the substrate can have a strong effect on them, the free-standing form of the structure is considered. Also, we ignore electron-electron interactions and thermal effects.

The Hamiltonians of the TB approach are implemented in the NEGF formalism to get the transmission spectra. The retarded Green’s function is given by^37^,

2 $$\:G\left(E\right)={\left[\left(E+\text{i}\eta\:\right)I-{H}_{C}-{\varSigma\:}_{SC}\left(E\right)-{\varSigma\:}_{DC}\left(E\right)\right]}^{-1}$$

where $\:E$ is the electron energy, **I** is the identity matrix, $\:\eta\:$ is an arbitrarily small positive number, $\:{H}_{C}$ is the central scattering region (device) Hamiltonian, and the self-energy for the source (drain) electrode is represented by $\:{\varSigma\:}_{SC\left(DC\right)}$. Details of this formalism can be found in^38^.

The spectral density operator is given by

3 $$\:{\varGamma\:}_{S\left(D\right)}\left(E\right)=\text{i}\left[{\varSigma\:}_{SC\left(DC\right)}\left(E\right)-{\varSigma\:}_{SC\left(DC\right)}{\left(E\right)}^{\dag }\right],$$

The electronic transmission probability (or the transport coefficient) can be calculated as

4 $$\:{T}_{e}\left(E\right)=\text{T}\text{r}\text{a}\text{c}\text{e}\left[{\varGamma\:}_{S}\left(E\right)G\left(E\right){\varGamma\:}_{D}\left(E\right)G{\left(E\right)}^{\dag }\right].$$

The local density of state (LDOS) for a specific atom (indicated by index *j*) can be evaluated as

5 $$\:\text{L}\text{D}\text{O}\text{S}{\left(E\right)}_{j}=\frac{-1}{\pi\:}\mathfrak{I}\left(G{\left(E\right)}_{j,j}\right),$$

where $\:\mathfrak{I}$ indicates the imaginary part. Net current from site *j* to *i*is evaluated by the following formula^39,40^

6 $$\:{I}_{i,j}\left(E\right)=\frac{4e}{h}\mathfrak{I}\left[{G}_{i,j}^{n}{\left(E\right)H}_{i,j}\right],$$

where $\:{G}_{i,j}^{n}\left(E\right)$ is an element of matrix $\:{G}^{n}\left(E\right)=G\left(E\right){\varGamma\:}_{S}\left(E\right)G{\left(E\right)}^{\dag }$. To study phononic properties, the force-constant method with the interactions up to four-nearest neighbors is used. The secular equation for phonons, which derives from Newton’s second law, reads^41^

7 $$\:DU={\omega\:}^{2}U,$$

with $\:U$ as the matrix containing the vibrational amplitude of all atoms, $\:\omega\:$ as the angular frequency, and $\:D$ as the dynamical matrix, which reads

8 $$\:D=\left[{D}_{i,j}^{3\times\:3}\right]=\left[\left\{\begin{array}{c}\frac{-{K}_{i,j}}{\sqrt{{M}_{i}{M}_{j}}}\:\text{f}\text{o}\text{r}\:\:j\ne\:i\\\:{\sum\:}_{j\ne\:i}\frac{{K}_{i,j}}{{M}_{i}}\:\text{f}\text{o}\text{r}\:\:j=i\end{array}\right.\right],$$

with $\:{M}_{i}$ as the mass of the $\:{i}^{th}$ atom, and $\:{K}_{i,j}$ represents $\:3\times\:3$ force tensor between the $\:{i}^{th}$and the $\:{j}^{th}$atoms, which is given by

9 $$\:{K}_{i,j}={U}^{-1}\left({\theta\:}_{i,j}\right){K}_{i,j}^{0}U\left({\theta\:}_{i,j}\right),$$

with $\:{\theta\:}_{ij}$ as the angle between the $\:{i}^{th}$and the $\:{j}^{th}$atoms. The unitary matrix $\:U\left({\theta\:}_{i,j}\right)$ is defined by the rotation matrix in a plane as

10 $$\:U\left({\theta\:}_{i,j}\right)=\left(\begin{array}{ccc}\text{c}\text{o}\text{s}{\theta\:}_{i,j}&\:\text{s}\text{i}\text{n}{\theta\:}_{i,j}&\:0\\\:-\text{s}\text{i}\text{n}{\theta\:}_{i,j}&\:\text{c}\text{o}\text{s}{\theta\:}_{i,j}&\:0\\\:0&\:0&\:1\end{array}\right),$$

with $\:{K}_{i,j}^{0}$ that is given by

11 $$\:{K}_{i,j}^{0}=\left(\begin{array}{ccc}{\phi\:}_{r}&\:0&\:0\\\:0&\:{\phi\:}_{{t}_{i}}&\:0\\\:0&\:0&\:{\phi\:}_{{t}_{o}}\end{array}\right),$$

where $\:{\phi\:}_{r},{\phi\:}_{{t}_{i}},{\phi\:}_{{t}_{o}}$are force constant parameters in the radial, in-plane, and out-of-plain directions of the $\:{j}^{th}$ atom, respectively. The $\:{D}_{D}$ matrix represents the dynamical matrix of the device section. For evaluation of the matrix elements one has to consider all 4NN effects in the summation, including atoms in the neighboring unit cells, i.e., $\:{D}_{i,j}={\sum\:}_{i}{\sum\:}_{j\in\:4NN}{K}_{{i}^{\text{D}\text{e}\text{v}\text{i}\text{c}\text{e}},{j}^{\text{D}\text{e}\text{v}\text{i}\text{c}\text{e}}}+{K}_{{i}^{\text{D}\text{e}\text{v}\text{i}\text{c}\text{e}},{j}^{\text{S}\text{o}\text{u}\text{r}\text{c}\text{e}}}+{K}_{{i}^{\text{D}\text{e}\text{v}\text{i}\text{c}\text{e}},{j}^{\text{D}\text{r}\text{a}\text{i}\text{n}}}$. Regarding coupling terms such as the elements of $\:{D}_{\text{D}\text{e}\text{v}\text{i}\text{c}\text{e}-\text{D}\text{r}\text{a}\text{i}\text{n}}$, the interaction between the first atom of the device and the first atom of the drain (i.e., diagonal elements) is already accounted. The force constants are taken from^42–44^, and are also given in Table 1.

The phononic transmission probability ($\:{T}_{ph}$) can be obtained by the Green’s function method, using $\:{\omega\:}^{2}$ instead of E in Eq. 2. Also, the phononic DOS (vDOS) can be evaluated using $\:\text{v}\text{D}\text{O}\text{S}\left({\omega\:}^{2}\right)=\frac{-1}{\pi\:}\mathfrak{I}\left[\text{T}\text{r}\text{a}\text{c}\text{e}\left(G\left({\omega\:}^{2}\right)\right)\right]$. The phononic transport is ballistic when $\:{L}_{D}$is much smaller than the phonon mean free path^41^, but it is also a function of the width of the GNR^45,46^. Since the system size is small, the assumption of ballistic transport seems reasonable.

**Table 1:** The TB and FC parameters^34,42,43,47^.

On-site energy (eV)	Fabric type
$\:0$	$\:-2.7$
Mass of the carbon atom ($\:{\varvec{M}}_{\varvec{C}}$)$\:\:\left(\mathbf{K}\mathbf{g}\right)$	$\:1.994\times\:{10}^{-26}$
Force-Constant parameters (N/m)	
$\:{\varvec{\phi\:}}_{\varvec{r}}^{1}$	$\:398.7$
$\:{\varvec{\phi\:}}_{{\varvec{t}}_{\varvec{i}}}^{1}$	$\:172.8$
$\:{\varvec{\phi\:}}_{{\varvec{t}}_{\varvec{o}}}^{1}$	$\:98.9$
$\:{\varvec{\phi\:}}_{\varvec{r}}^{2}$	$\:72.9$
$\:{\varvec{\phi\:}}_{{\varvec{t}}_{\varvec{i}}}^{2}$	$\:-46.1$
$\:{\varvec{\phi\:}}_{{\varvec{t}}_{\varvec{o}}}^{2}$	$\:-8.2$
$\:{\varvec{\phi\:}}_{\varvec{r}}^{3}$	$\:-26.4$
$\:{\varvec{\phi\:}}_{{\varvec{t}}_{\varvec{i}}}^{3}$	$\:33.1$
$\:{\varvec{\phi\:}}_{{\varvec{t}}_{\varvec{o}}}^{3}$	$\:5.8$
$\:{\varvec{\phi\:}}_{\varvec{r}}^{4}$	$\:1$
$\:{\varvec{\phi\:}}_{{\varvec{t}}_{\varvec{i}}}^{4}$	$\:7.9$
$\:{\varvec{\phi\:}}_{{\varvec{t}}_{\varvec{o}}}^{4}$	$\:-5.2$

## Results and discussion

### Effect of outer radius

**Fig. 2:**
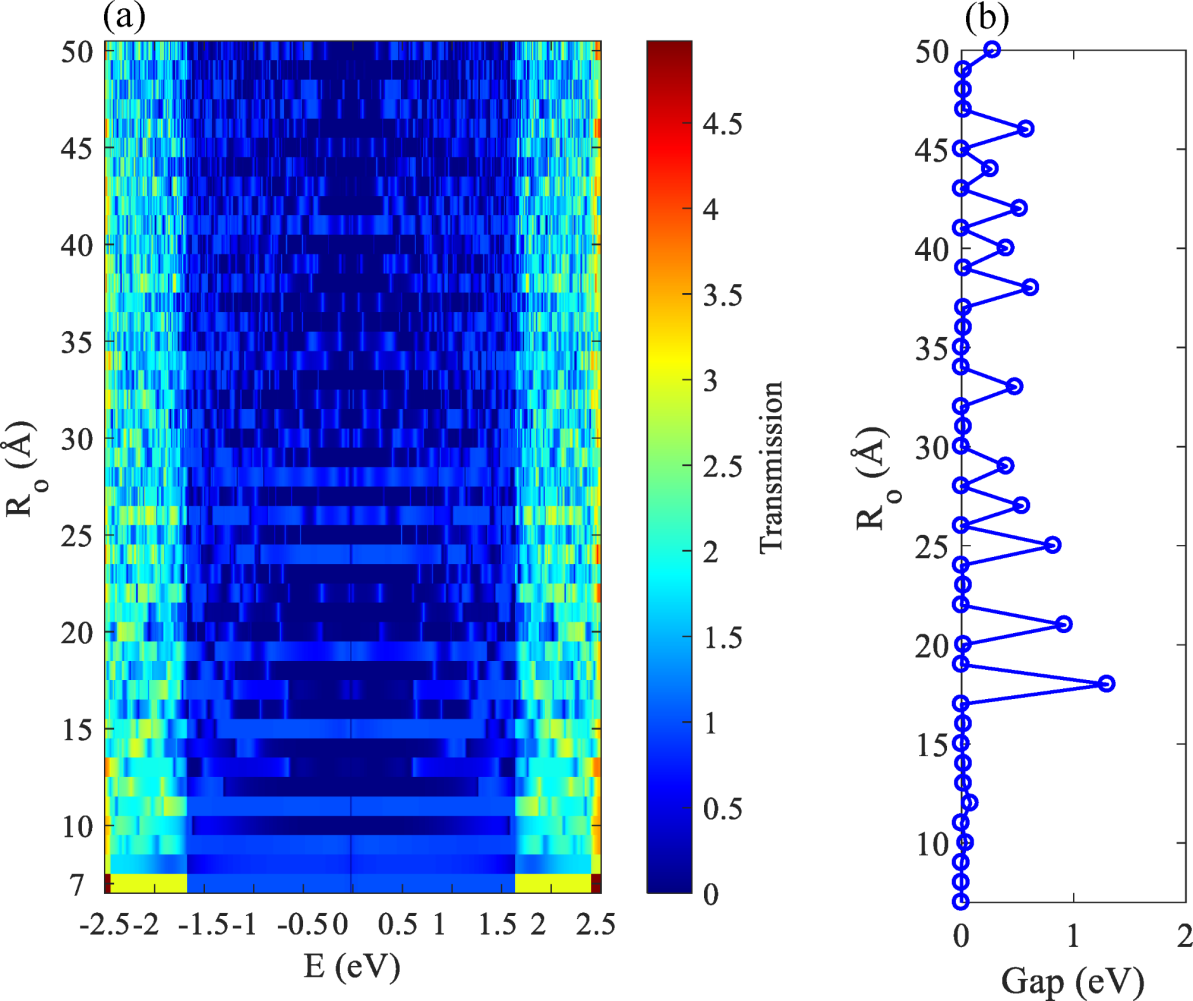
(**a**) The transmission coefficient, and (**b**) the transport energy gap as a function of $\:{\text{R}}_{\text{o}}$.

In a first step, we study the effect of the outer radius on the electronic transmission spectrum (Fig. 2(a)), or on the other hand, we consider first a circular graphene nanodisk. As one can see, the leads draw the main limitation of the transport properties^48^, note the blue shade between E~$\:\pm\:1.7$ eV in the transmission spectrum which is stretched from $\:{\text{R}}_{\text{o}}$=7 Å to larger radii. The transport coefficient is one for this energy range. This range is related to the bands that arise from the zigzag edge geometry and ends by reaching the Fermi energy. The simple TB model for graphene is fitted to describe the most important bands close to the Fermi energy. Therefore, it is reasonable to consider this energy range as a reference in this study. However, since the simple TB model cannot reproduce other electronic bands of the graphene, it is more reasonable to discuss the energy ranges close to the Fermi energy for larger devices. The transport energy gap ($\:{\text{E}}_{\text{g}\text{a}\text{p}}$) is also investigated in Fig. 2 (b). The dependence of $\:{\text{E}}_{\text{g}\text{a}\text{p}}$ on the outer radii is irregular and depends on the specific atomic configuration that appears when the size of the device is changed. Remarkably there is no direct correspondence between the transport and energy gaps, e.g., for $\:{\text{R}}_{\text{o}}$=10 Å one can see that the transmission function shows drastic reduction (dark blue), however, the $\:{\text{E}}_{\text{g}\text{a}\text{p}}$ is very small.

**Fig. 3:**
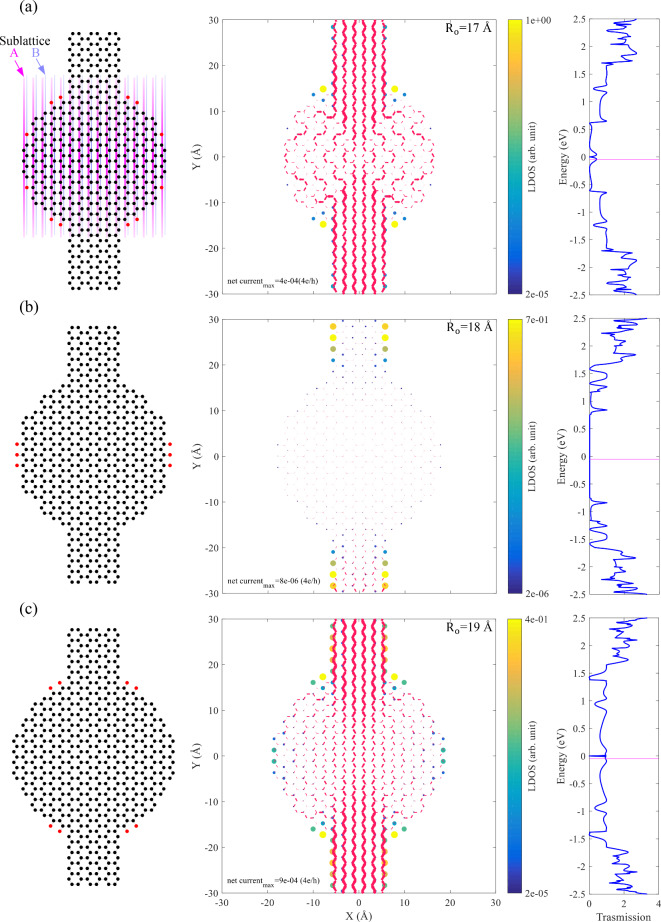
The atomic configurations with Klein edges are marked in red (left), the LDOS and net current at E=−0.05 eV (middle), and the electronic transmission function (right) for a graphene nanodisk with an outer radius of (**a**) 17 Å, (**b**) 18 Å, and (**c**) 19 Å.

For a further investigation, we choose outer radii of 17, 18, and 19 Å and plot the LDOS together with the net current at E=$\:-$0.05 eV, indicated by the magenta line in the transmission spectrum in Fig. 3. The outer edge geometry is a mixture of Klein, zigzag and armchair edges. Figure 3 (a) show a nanodisk with $\:{\text{R}}_{\text{o}}$=17 Å. There are two Klein edge atoms near each side of the leads (belong to different sublattices), and the high LDOS indicates a strong localization, which helps the extension of the electrodes into the nanodisk, see the local current that doesn’t feel the nanodisk structure up to about $\:\pm\:$10 Å in the y-direction. The transmission spectrum (Fig. 3(a), right panel), shows that the transport coefficient is degraded. This degradation can be attributed to the electronic scattering of the device due to the presence of other edges. As reported in^28^, the cove and cape type edges have different localization characteristics with different extension into the structure. Also, zKLs on both sides of a nanostructure belong to different sublattices, causing mixing and splitting of the energy bands associated with them. This is shown in the left panel of Fig. 3(a), where the two sublattices are indicated by distinct colors in vertical lines. Such edges can also affect the transmission spectrum. Furthermore, it is shown that the presence of Klein edges on a sublattice is of great importance in nanoribbons^28^. Moreover, in each side of the disk (we mean left or right side), one can see that if the aKLs and zKLs are present simultaneously, they belong to different sublattices, which makes it possible for hybridization. We should note again that the observation of both zKL and aKL are reported in the experiment^49^.

For $\:{\text{R}}_{\text{o}}$=18 Å the transmission spectrum shows a semiconducting behavior, see Fig. 3 (b). The edge of this outer radius is very different from $\:{\text{R}}_{\text{o}}$=17 Å, containing only zKLs. From the transmission spectrum one can conclude that the electronic configuration of the device in the transport energy gap is not matched with the leads. As mentioned in Refs^28,50^, the zKLs belonging to different sublattices allow the mixing and splitting of energy bands in a periodic system that is different from the ZGNRs. While flat bands arising from localized zigzag edges appeared for k > 2π/3 (k is the wave vector), adding zKLs changes the situation so that flat bands are already present from k = 0 to k < 2π/3^28^. This can be a possible explanation for the semiconducting behavior of the system.

The structure of the case with $\:{\text{R}}_{\text{o}}$=19 Å (Fig. 3 (c)) is similar to that of the case with $\:{\text{R}}_{\text{o}}$=17 Å, except for the absence of zKLs. In this system, the electronic transport coefficient is not degraded as it is in the case with $\:{\text{R}}_{\text{o}}$=17 Å. The disk can be divided the into four quarters to verify the occurrence of the aKLs respect to the two sublattice of the graphene. It can be seen that the two quarters in the bottom, host aKLs on different sublattices, while aKLs in the two quarters on the left or right side belong to the same sublattice. From the LDOS map, one can see that the further aKLs (respect to the electrode edge) have lower LDOS, suggesting that the proximity of aKLs to the electrodes is important for preserving the transmission probability. Figure S.3 shows atomic structures with vertical lines indicating the sublattices. Similar figures are plotted in the SM for the structures shown in the manuscript, see Figures S.4 and S.5.

**Fig. 4:**
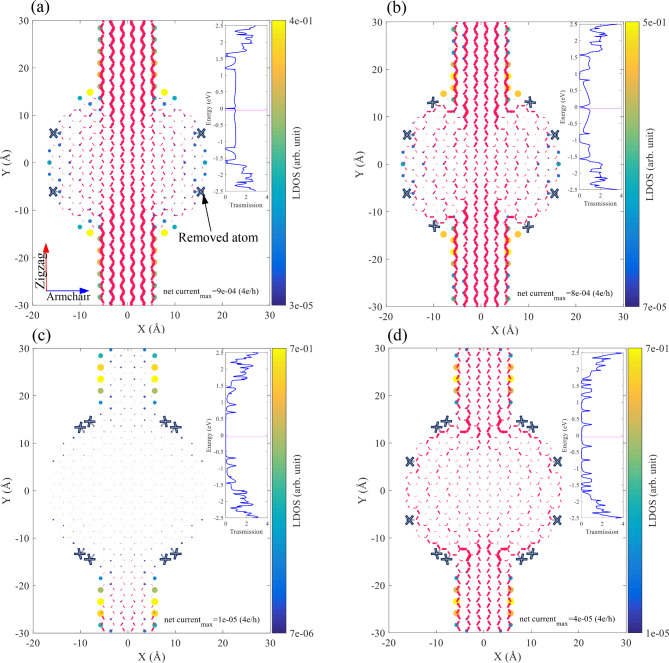
The net current (indicated by the magenta arrows) and the local density of states corresponding to the transmission probability at E=$\:-$0.05 eV (indicated by the magenta line in the insets) for the case of (**a**) omitted zKLs (indicated by the gray crosses) and keeping aKLs, (**b**) removing zKLs and keeping four of aKLs, (**c**) removing all aKLs and keeping zKLs, and (d) removing all Klein edges. The insets show the corresponding transmission spectra as a function of energy. The outer radius for all panels is 17 Å. The arrows are scaled in both length and thickness.

We chose an outer radius of 17 Å, which is an interesting case since it has both Klein edges with armchair and zigzag edges to further investigate how the Klein edges can impact the transmission probability. We investigate four configurations based on the $\:{\text{R}}_{\text{o}}$=17 Å (Fig. 4), by removing the Klein edges and studying the resulting electronic transport properties. Based on the experimental report in^51^, Klein edges are also observed in the armchair direction. Moreover, a combined experimental and theoretical study shows the possibility of selective hydrogenation of AGNRs, which can resemble the existence of aKLs on the same or on different sublattices^18^.

We first remove four zKLs from the left and right sides of the structure, the removed atoms are marked with gray bold crosses (Fig. 4 (a)). The Klein edges near the electrodes which are aKLs, can effectively extend the electrode(s) into the disk. By removing zKLs, the transmission spectrum becomes similar to that of the electrodes in a wide energy range, see the inset of Fig. 4 (a). Matching between electronic structures of the electrodes and the device determines the transmission probability. As mentioned earlier, zKLs in a graphene-based nanostructure on both sides can produce dispersive bands in a periodic system near the Fermi energy, which affects the electronic structure of the device, creating a mismatch between the device and electrodes.

Removing four more aKLs from the system lowers the transport coefficient (Fig. 4(b)). The transmission coefficient shows evidence of the Fano resonance, which also occurred in the previous system. The Fano resonance can be identified in the transmission probability when it shows an asymmetric behavior, resulting from the coupling of a localized state with the energy continuum of states^52^. So far, we see that a higher number of aKLs is more effective in keeping the transmission spectrum close to that of the electrodes. Removing all aKLs and keeping zKLs strongly affects the transport coefficient and makes the system a semiconductor with $\:{\text{E}}_{\text{g}}$>1.2 eV (Fig. 4 (c)). We have already discussed how zKLs can change the electronic configuration of the system, increasing the mismatch between the leads and the device. Removing all Klein edges in Fig. 4 (d), results in a high suppression of the transport probability, yet the system is metallic. The devices of Fig. 4 (a) and (c) can also be studied in the periodic configuration, see Figure S.6 for the band structure and probability amplitude for three selected states. The system shown in Fig. 4 (a) possess a good performance in transmitting electronic states from source to drain. The electronic band structure of the periodic configuration of the device shows that the bands are dispersive, indicating that the zigzag edge states are hybridized with the states in the disk, see Figure S.6 (a). However, for the case shown in Fig. 4 (c), the band structure shows flat bands which accompanying the probability amplitude of the marked states, indicating that they are not hybridized, see Figure S.6 (b).

It is already mentioned that the aKLs on the sides (left and right) of the disk belong to different sublattices, which raises the question what is the role of the disk symmetry? Is the belonging of the aKLs to different sublattices crucial for the appearance of such a behavior? We have removed the right side of the disk, and it is observed that the overall behavior remains similar to that of obtained for the disk (see Figure S.7), suggesting that the role of aKLs is more related to belonging to the same sublattice.

**Fig. 5:**
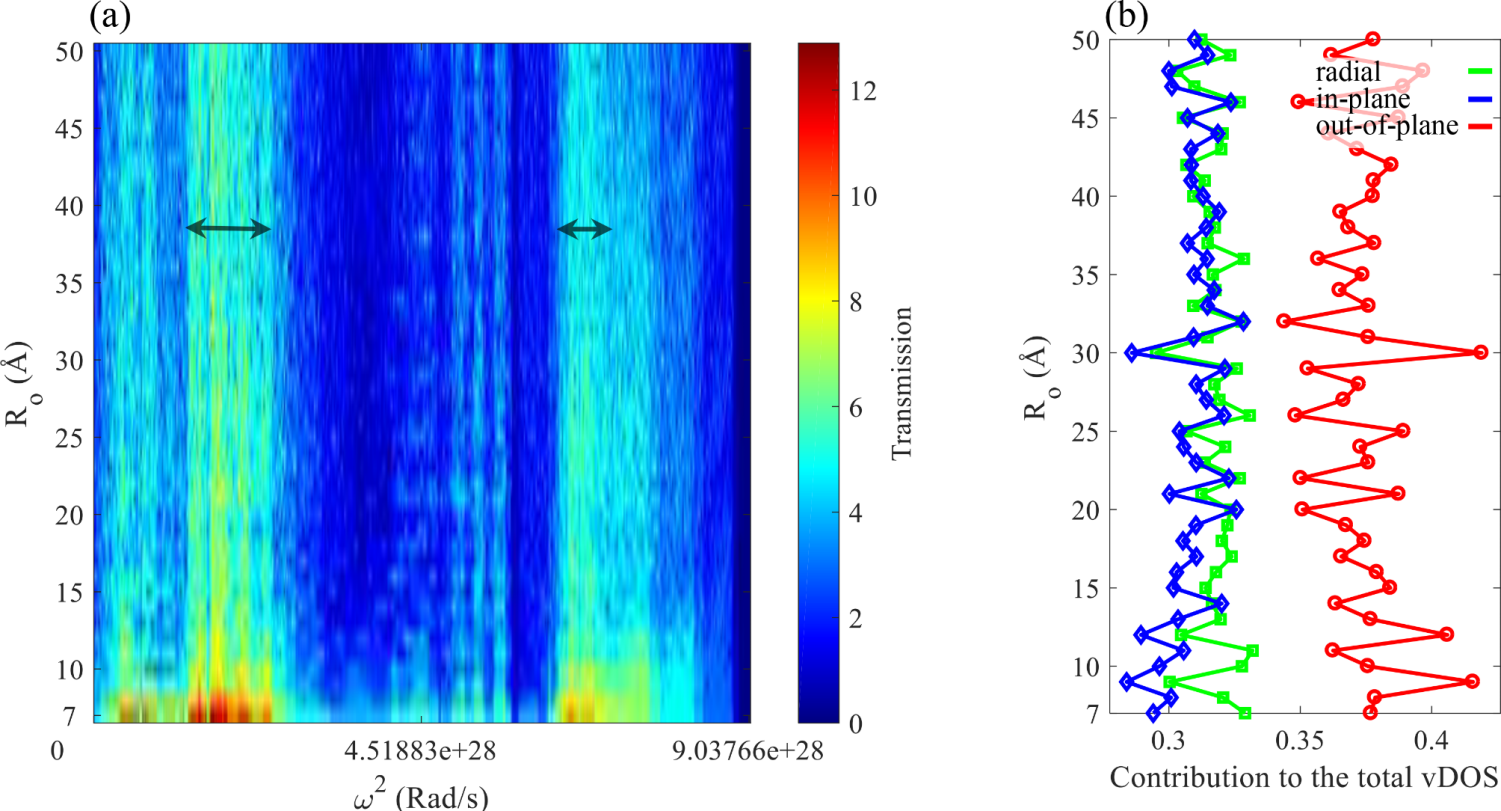
(**a**) The vibrational transmission function, and (**b**) the contribution of the radial (green), in-plane (blue), and the out-of-plane (red) components of phonons as a function of $\:{\text{R}}_{\text{o}}$.

The size effect of the device should also be considered. As the size increases, the effect of the edges is expected to become insignificant. However, in the system studied up to outer radii of 50 Å, the electronic transmission spectrum does not converge to a spectrum. For the last step in this section, we examine the phononic transport coefficient as a function of frequency and the outer radii in Fig. 5 (a). As it is expected, increasing the device size introduces more phonon energy levels that do not necessarily match with those of the electrodes, resulting in a complicated transport coefficient. This figure suggests that some frequency regions are less affected by increasing the device size, such as those of marked with double-sided black arrows in Fig. 5 (a). As the size increases, the number of uncoordinated atoms in the edges also increases, which can drastically affect the available vibrational levels. This becomes more important for phonons, where interactions up to 4NN are essential to describe the phononic behavior of graphene using the FC method. However, we do not find a regular relation between Klein edges, and in general the edge geometry of the circular disk and the phononic transmission spectrum. We have also studied the contribution of the in-plane, out-of-plane and radial components of the phonons to the total vDOS in Fig. 5 (b), which shows that the surface of the nanodisk can possess different sensitivities in terms of phonon-related properties.

### Effect of inner radius

**Fig. 6:**
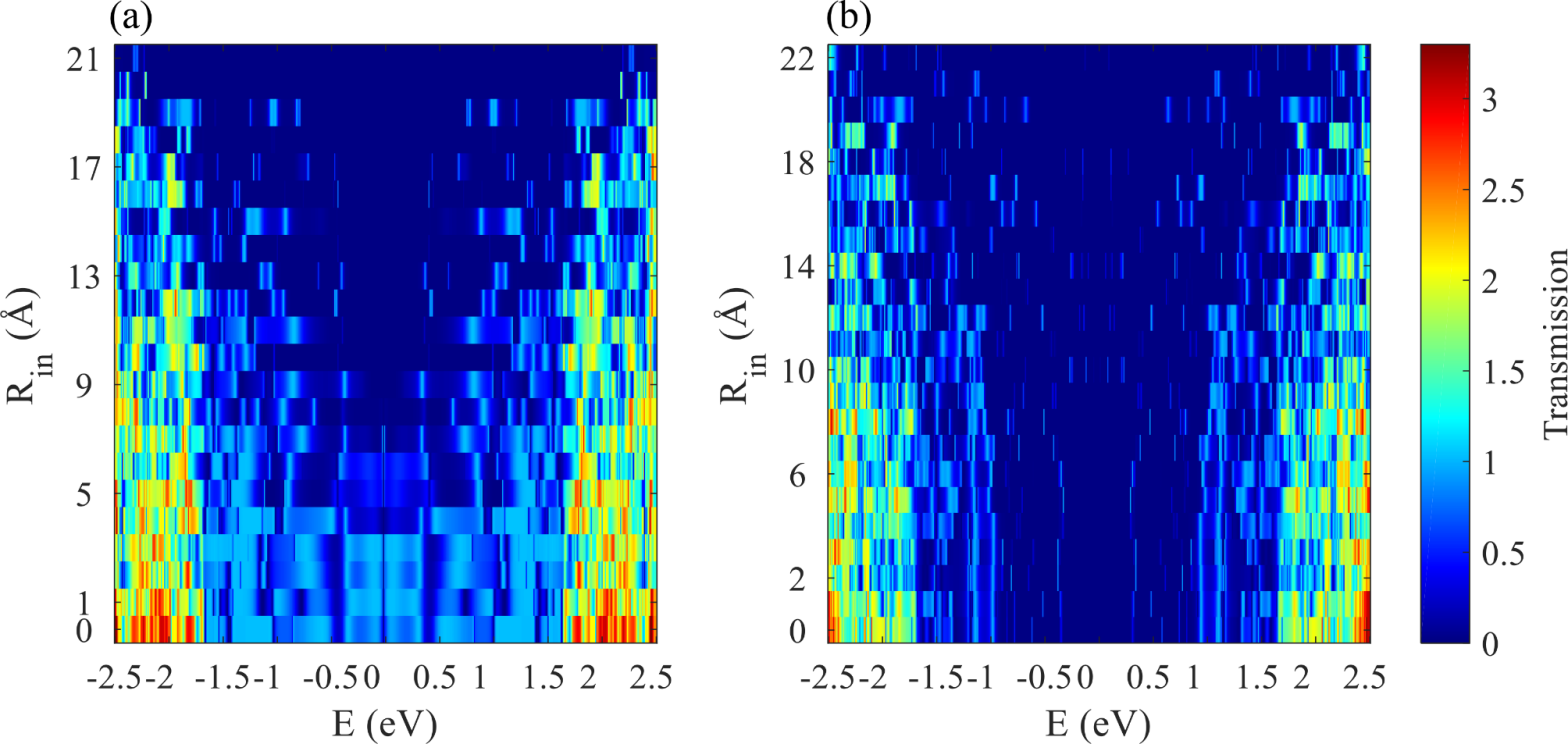
The electronic transmission function as a function of $\:{\text{R}}_{\text{i}\text{n}}$ for an outer radius of (a) 26 Å and (b) 27 Å.

Among 44 different simulated nanodisk configurations, we select two outer radii, one being an ultra-narrow bandgap semiconductor with aKLs ($\:{\text{R}}_{\text{o}}$=26 Å) and the other being a semiconductor with zKL edges ($\:{\text{R}}_{\text{o}}$=27 Å), for further investigation. The effect of the outer radius and the effect of different Klein edges were examined in the previous section. Here, the behavior of the inner radius appears to be more complex. Note that the inner and outer edge states can couple across the bulk of the system, depending on the sublattices involved^53^. Figure 6 illustrates that in very small inner radii, the transmission spectrum shows a behavior close to that of the corresponding disk. Forming a hole in the disk can create a variety of edges. These edges may or may not belong to the same sublattice, which strongly changes the electronic configuration of the device. The center of the coordination system is the same for all studied structures, i.e. the edge geometry for a given $\:{\text{R}}_{\text{i}\text{n}}$ is independent of $\:{\text{R}}_{\text{o}}$. A disk with $\:{\text{R}}_{\text{o}}$=26 Å is a metallic system, while a nanodisk with an outer radius of 27 Å is a semiconductor with $\:{\text{E}}_{\text{g}}$~0.8 eV.

It is well known that electrons in graphene ribbons exhibit different behaviors depending on the type of edge and the width of the ribbon^9,53^. One possible expectation is that, a smaller width of the annulus (ring) can result in a stronger electronic confinement, but a ring structure made up of graphene is not as smooth as an annulus. On the other hand, rings of the same width for different inner or outer radii are not necessarily similar in terms of edge geometries. Such structures have irregular boundaries. In ring structures, due to the irregular boundary [54] the electronic behavior can be different from the well-studied nanostructures such as armchair and zigzag edges. For example, the energy gap of R_in_=21 Å is smaller than for larger outer radius for R_o_=26 Å, which means that the transport energy gap for rings of the same width with different R_o_ are different, indicating a complex electronic structure arising from the irregular edge geometries, which needs to be carefully studied for each particular case. One interpretation is that the electronic states of the device and the electrodes are matched in specific energies. On the other hand, the sharp peaks in the transmission spectrum show that the device has states that match those of the electrodes in a limited energy range, revealing the highly localized nature of such states.

### Test models

In this subsection, we present some models to investigate a specific purpose. First, it is crucial to summarize our findings:

-If a device contains only zKLs, then the strong suppression of the transport coefficient can lead to a semiconducting system.
-If the device contains both aKLs and zKLs, the transport coefficient is highly reduced in the energy region close to the Fermi energy due to the coupling between them.
-If the device contains only aKLs and they are close to the electrodes, the transport coefficient becomes close to the maximum possible value in the transport configuration, which is that of the electrodes.

**Fig. 7:**
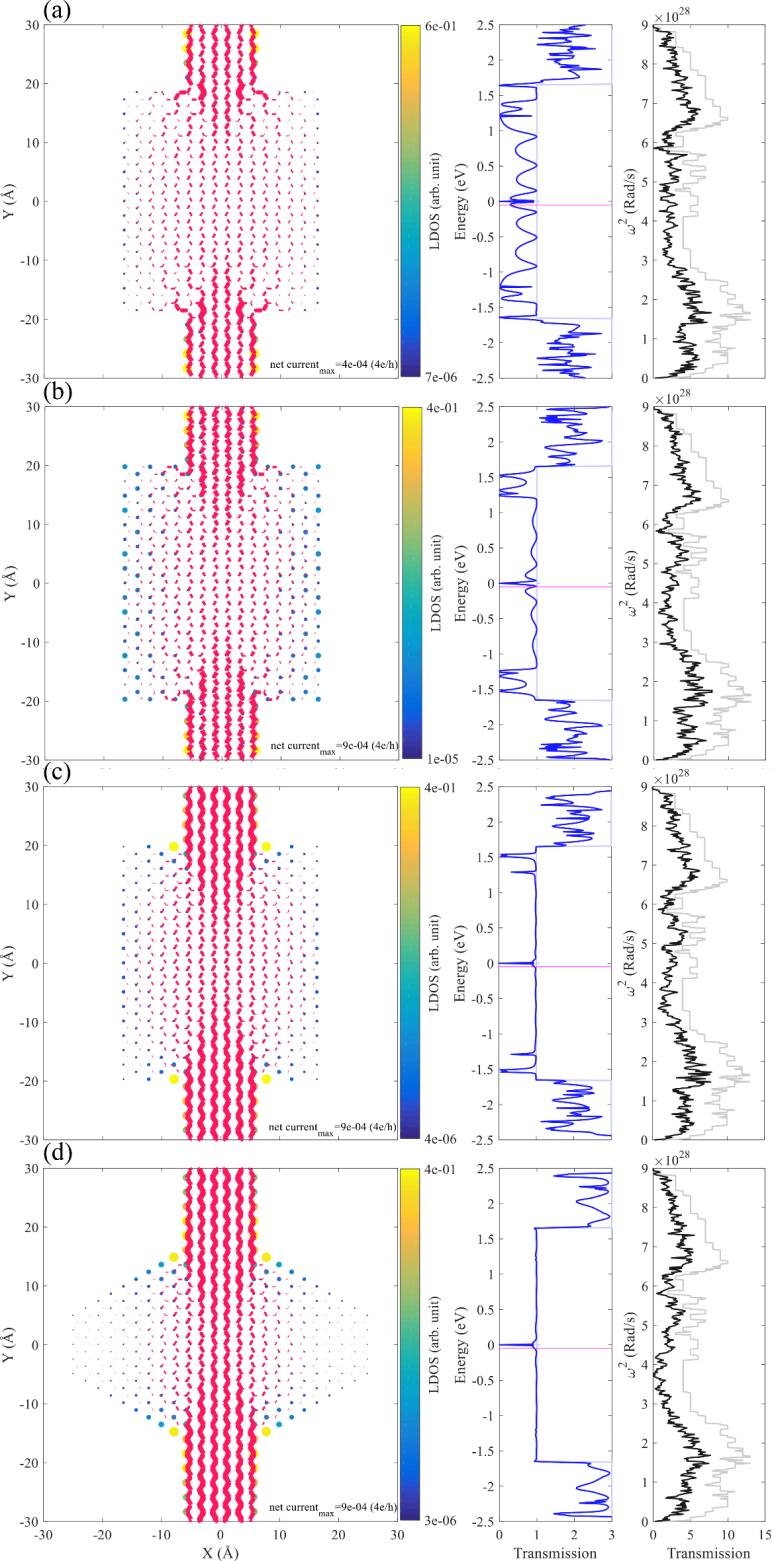
The LDOS and the net current at E=$\:-$0.05 eV (left panel), the electronic (the middle panel), and the phononic (right panel) transmission functions of a rectangular graphene quantum dot (a) without single bonded atoms, (b) with aKLs at the corners, (c) with additional aKLs, and (d) the double trapezoidal graphene quantum dot with the maximum number of aKLs. The pale colors in the transmission spectrum indicate the transport coefficient of the electrodes. The magenta line is the energy for which the LDOS and the net current are calculated.

We test the above statements in four cases. The first three cases are rectangular quantum dots, and the last case is an irregular hexagonal (or two trapezoidal) quantum dot, all connected to two ZGNR electrodes with 12 atoms in the width (12-ZGNR). The right panel in Fig. 7 (a) shows a rectangular graphene quantum dot with no single bonded atoms. As one can see, the transmission spectrum (Fig. 7 (a), middle) shows a suppressed transport coefficient with wide curves, as well as sharp thin peaks, suggesting that resonant tunneling is involved in the electronic transmission. Careful investigation of the quantum dot reveals that at the four corners of the rectangle there are atoms belonging to the same sublattice, as well as armchair edges. All these can affect the electronic configuration of the device, which is reflected in the transmission spectrum, see the left panel of Fig. 7 (a) for the LDOS and the net current at E=$\:-$0.05 eV. The phononic transmission spectrum (black solid line in Fig. 7 (a), right panel) shows a reduction compared to that of the electrodes (gray solid line). Extending the device by one dimer line in each direction along the transport direction allows the appearance of some aKLs at the corners, Fig. 7 (b). The transmission function shows shallow curves, indicating a better match between the device and the electrodes. However, the phononic transport coefficient is reduced. By adding more aKLs to the rectangular quantum dot (as shown in Fig. 7 (c)), the electronic configuration of the device is better matched to the electrodes. This is evident from the transmission spectrum (middle panel). The aKLs closest to the electrodes have the highest LDOS at the specified energy. However, the phononic transmission function is reduced and shows no clear behavior with respect to the addition of Klein edges.

As of the last model, we proposed a case with edges that almost consists of aKLs (Fig. 7 (d)) to improve the matching of the electrodes and the scattering region, from the electronic properties point of view. The electronic configuration of the system is confirmed to be exceptionally well matched based on the transmission spectrum. The phononic transmission spectrum shows a significant reduction due to the increased number of undercoordinated atoms compared to previous cases.

## Summary

The effect of the outer radius of circular graphene structures, formed by growing a disk on a fixed-length zigzag nanoribbon in the device region, on the phononic and electronic transmission spectra has been investigated using the TB and FC methods implemented in the NEGF formalism. The numerical results suggest that armchair Klein edges near the electrodes in small disks can help to preserve the electronic transmission spectrum, while zigzag Klein edges suppress the electronic transport coefficient. There is no clear evidence for the effect of Klein edges on the phononic transport coefficient. We tested our findings by designing graphene-based quantum dots with and without aKLs. The results show the importance of aKLs in preserving the electronic transport coefficient. This seems to be rooted in the same sublattice occurrence of the aKLs. The independence of electronic and phononic transport coefficients can be useful in thermoelectrics. This is because reducing the lattice thermal conductivity and maintaining the electronic transport coefficient are both important for improving thermoelectric performance.

Due to the irregular boundaries in a ring structure, the effect of the inner radius is complicated. Roughly speaking, the outer radius primarily determines the nature of the transport system, and changing the inner radius can change the transport gap.

The details of the edges of the devices affect significantly their electronic properties. The edges produce complex effects on the electronic structure, implying that for sensor applications, one should consider the effects of the Klein edges.

## Electronic supplementary material

Below is the link to the electronic supplementary material.

Supplementary Material 1

## Data Availability

Data is provided within the manuscript or supplementary information files.
