# Supplementary material for: Influence of Klein edges on Phononic and electronic transport in circular graphene devices
Source: Sci Rep. 2024 Nov 25;14:29196. doi: 10.1038/s41598-024-80746-y (PMC11589862; doi:10.1038/s41598-024-80746-y)
Supplement: Supplementary file 1 — Supplementary Material 1 [file 41598_2024_80746_MOESM1_ESM.pdf]

Supplementary Material

Influence of Klein Edges on Phononic and Electronic Transport in Circular Graphene Devices

M.Amir Bazrafshan[1], Farhad Khoeini*, [1], Bartłomiej Szafran[2]

[1]*Department of Physics, University of Zanjan, P.O. Box 45195-313, Zanjan, Iran*

[2]*AGH University of Krakow, Faculty of Physics and Applied Computer Science, al. Mickiewicza 30, 30-059, Krakow Poland*

Corresponding author: Farhad Khoeini, khoeini@znu.ac.ir

The detailed configuration of the radii considered in this work is shown in Figure S.1. Each radius step is indicated by a different color. For better recognition of the edge structure, the colors between the concentric circles and the corresponding atoms considered in that radius step are preserved.

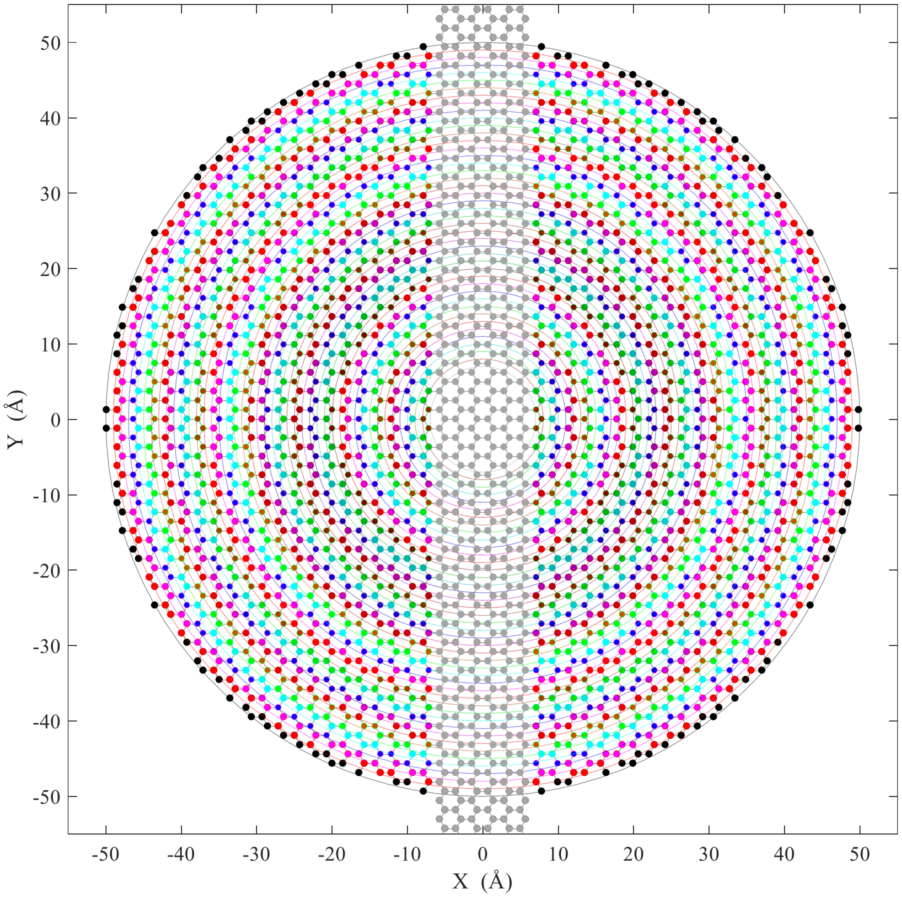

Figure S.1. A representation of the radii considered in the paper. Each circle represents a radius of either the nanoring or the nanodisk. Colors are preserved at each step of the radius change.

Figure S.2 shows the transmission coefficient for R_o=41 Å (a) and R_o=46 Å (b).

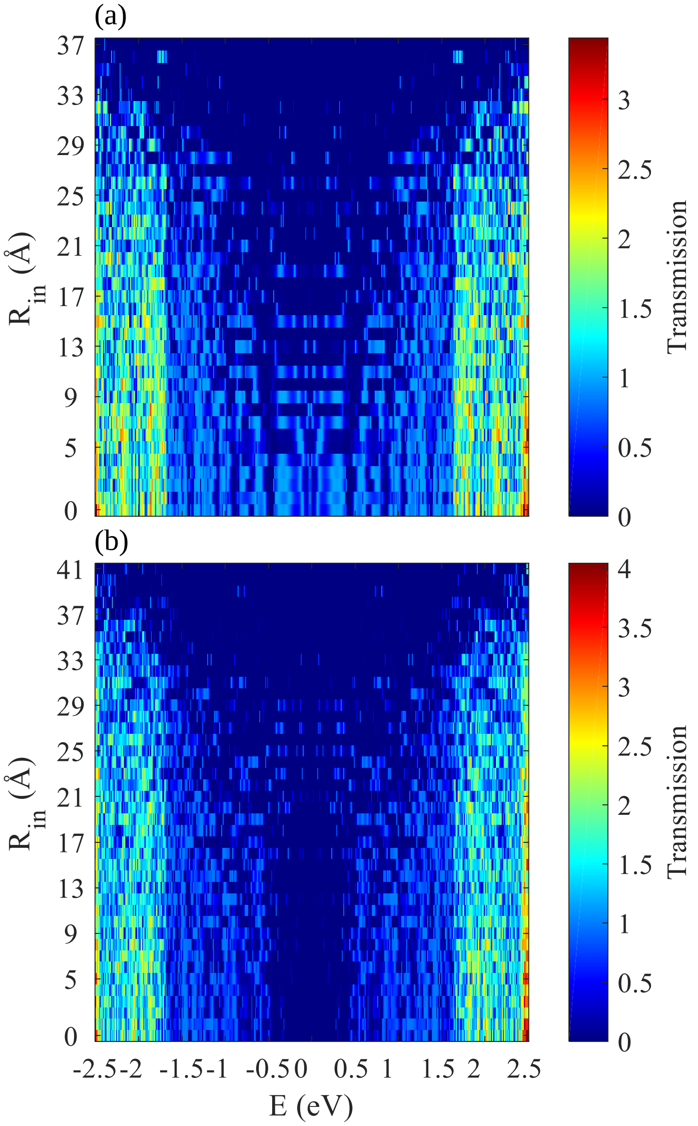

Figure S.2. Transmission coefficient for (a) R_o=41 Å, and (b) R_o=46 Å for various inner radii.

The transmission spectrum versus energy and the atomic structures with vertical lines indicating sublattices of Figures 3, 4, and 7 of the manuscript, shown in Figures S.3, 4, and 5, respectively. The Klein edges are marked with red filled circles.

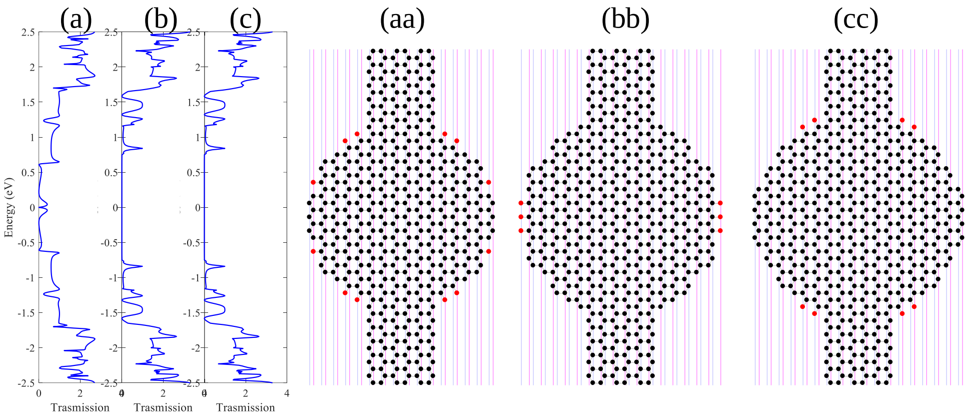

Figure S.3. The transmission functions and the corresponding atomic configurations with vertical lines indicating sublattices, according to Figure 3 of the manuscript. The Klein edges are marked with red filled circles.

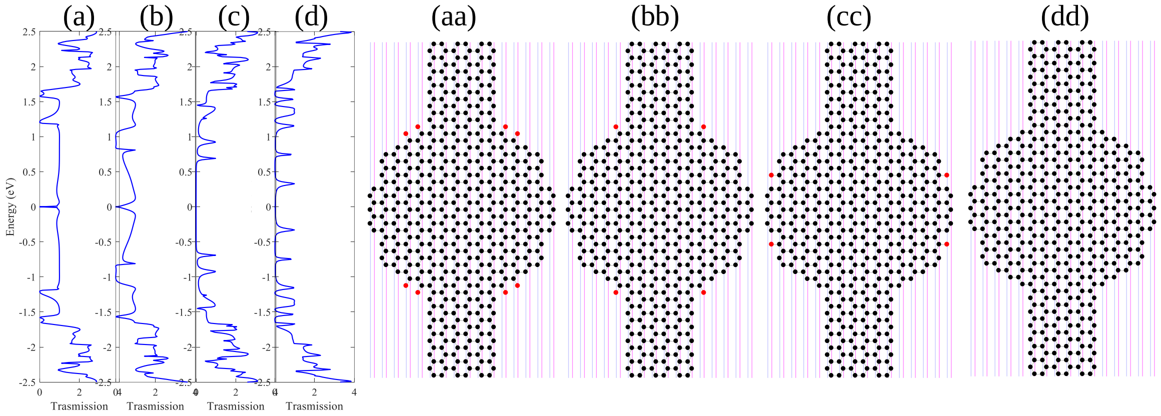

Figure S.4. Same as Figure S.3, but for Figure 4 of the manuscript.

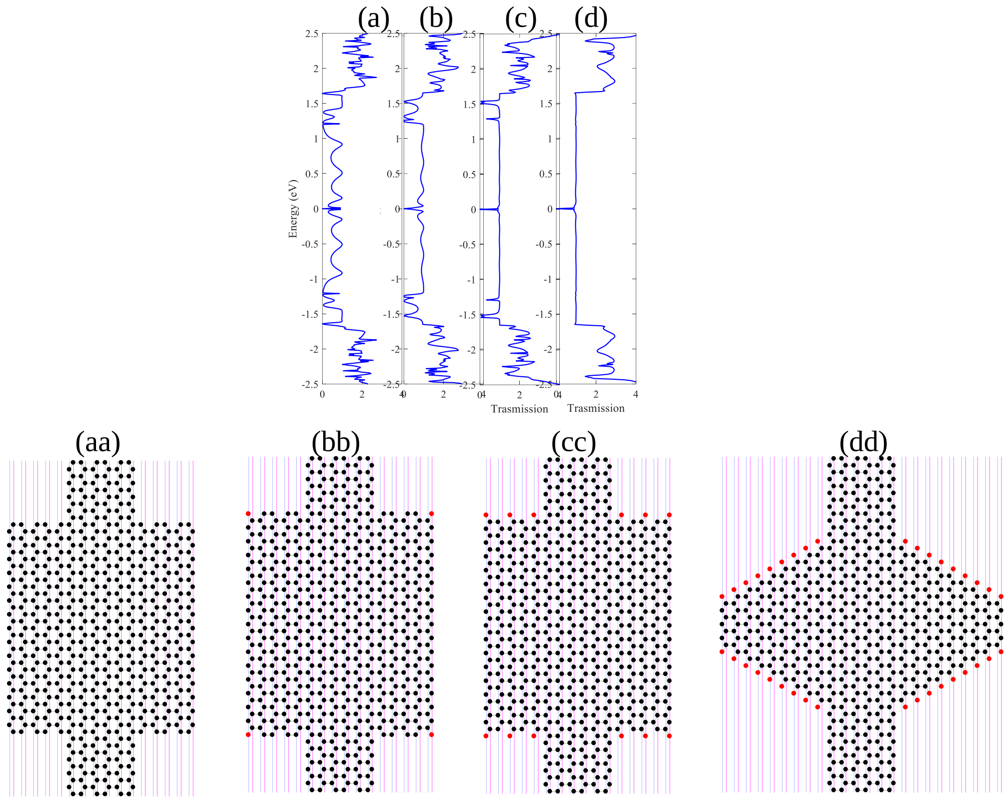

Figure S.5. Same as Figure S.3, but for Figure 7 of the manuscript.

The electronic band structure and probability amplitude of the marked states (with red circles) for selected devices in the periodic configuration are shown in Figure S.6.

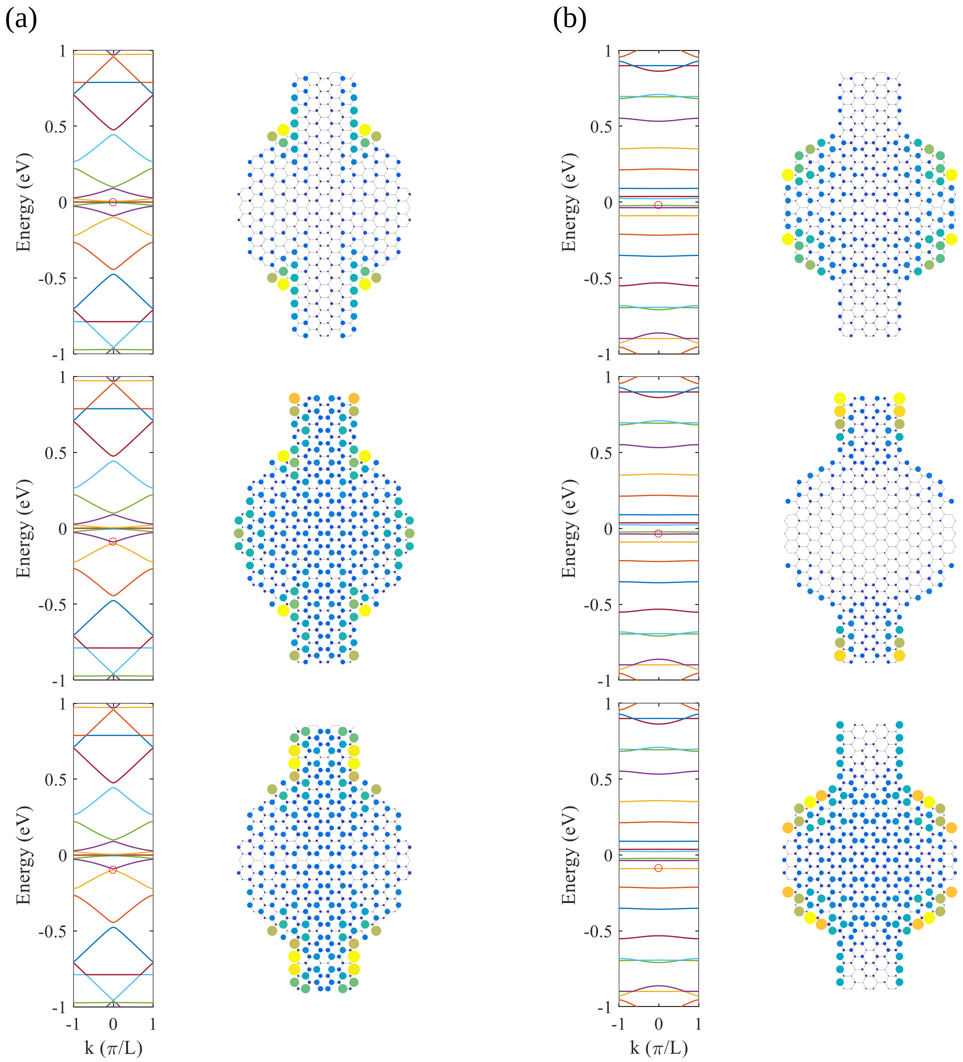

Figure S.6. The electronic band structure and probability amplitude of three selected states, marked by red circles, of the systems shown in Figure 4(a) and (c) of the manuscript, here shown in (a) and (b), respectively. To obtain the band structure, the device is considered in the periodic boundary condition.

Some possible edge configurations of the case with R_o=17 Å, with cutting one side of the disk together with the net current and the LDOS maps (Figure S.7).

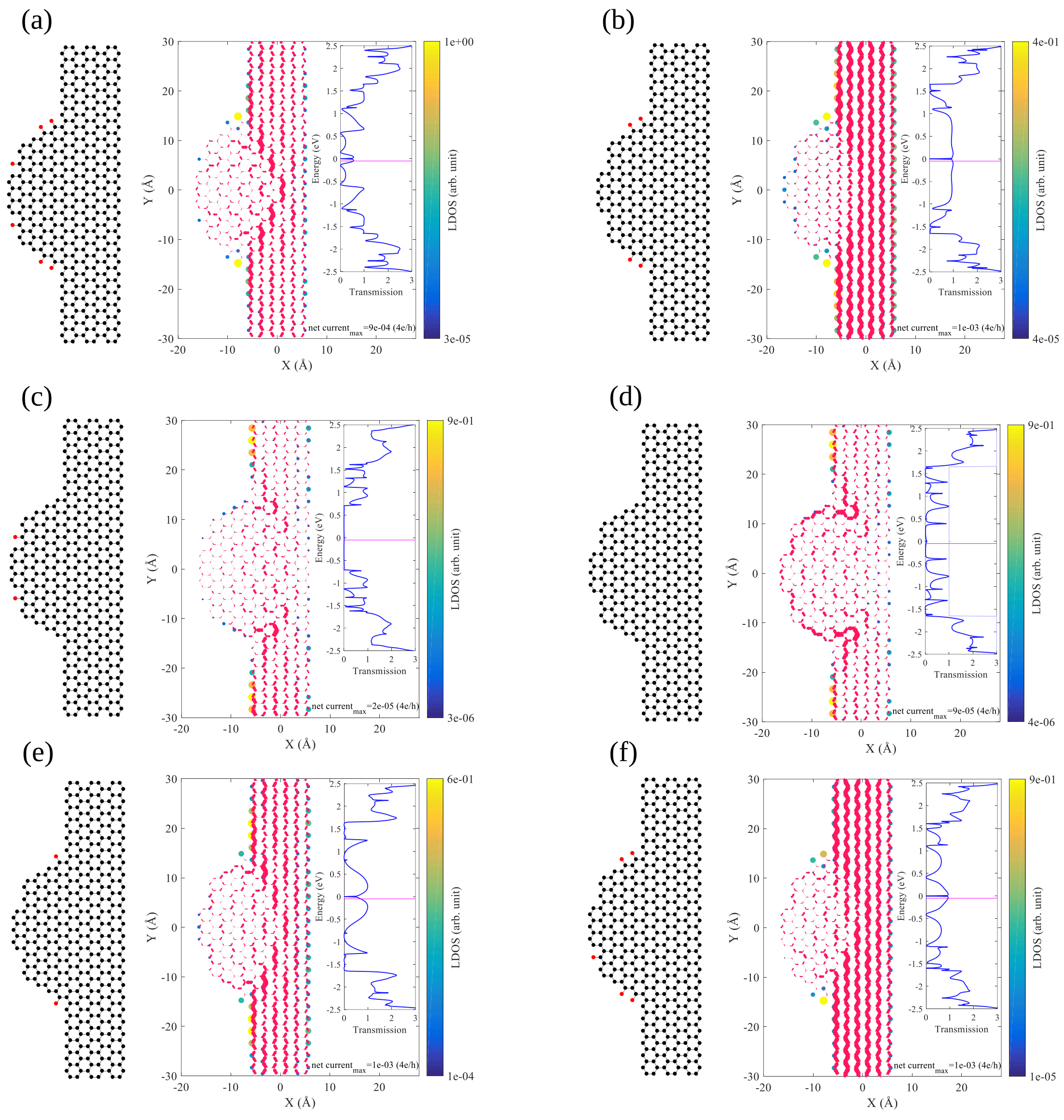

Figure S.7. The net current and LDOS mapped onto the atomic structures at E=−0.05 eV for the one-sided disk geometries with R_o=17 Å in the cases where (a) no Klein edges are removed, (b) the zKLs are omitted, (c) the zKLs are present while the aKLs are removed, (d) in the absence of Klein edges, (e) only the two aKLs closest to the electrodes are present, and (f) with the removal of only one zKL. The left panels illustrate the presence of Klein edges (marked in red) in the system. The electronic transmission probability of the system is shown in the inset. The magenta line indicates the energy at which the net current and LDOS belong.
